# Creativity, Eye-Movement Abnormalities, and Aesthetic Appreciation of Magritte’s Paintings

**DOI:** 10.3390/brainsci12081028

**Published:** 2022-08-03

**Authors:** Lindsey M Ward, Zoi Kapoula

**Affiliations:** IRIS Lab, Neurophysiology of Binocular Motor Control and Vision, CNRS UAR 2022 Neurosciences, UFR Biomedical, University of Paris, 45 Rue des Saints Pères, 75006 Paris, France; lward@mednet.ucla.edu

**Keywords:** saccades, vergence, eye movements, surrealism, Magritte, art, dyslexia, creativity

## Abstract

Dyslexic children have been shown to be more creative than their non-dyslexic counterparts. They have also been shown to have an abnormal oculomotor profile while viewing targets in free space, making vergence or saccadic eye movements while reading or when viewing Op art. They show a slower deceleration of their eye movements and a difficulty in coordinating their two eyes to obtain single fused vision in depth. Interestingly, their abnormal oculo-motor profile is exacerbated while reading more difficult texts. Given these differences, we postulate that dyslexics’ increased creativity may be related to their different eye movement control affecting how they perceive the world. Therefore, we decided to measure adolescent dyslexics’ creativity, oculomotor profile, and subjective responses while they viewed three paintings by Magritte. These were chosen to stimulate the perception of hidden conceptual spaces or stimulate conflict between the perception of the figural and textural content. For the first time to our knowledge, dyslexic adolescents were demonstrated to be more creative in terms of flexibility and fluidity than their non-dyslexic peers. Subjectively, while viewing the Magritte paintings, dyslexics reported fewer conceptual spaces and fewer hidden words than their non-dyslexic peers; thus, they confabulated less than non-dyslexics. Dyslexics also demonstrated an abnormal oculomotor profile similar to those that we have shown when reading, viewing randomized targets, and while perceiving illusions of depth in Op art paintings, in that they demonstrated difficulty with disconjugation and abnormalities in their eye velocity profiles. We propose there may be a link between dyslexic increased creativity and their eye movement abnormalities. Similar to reading nonsense text, we propose that Magritte’s contradictory paintings exacerbate dyslexics’ eye movement abnormalities. These eye movement abnormalities while viewing these particular paintings might provide a physiological signature suggesting a contribution of their unusual eye control to their higher creativity scores.

## 1. Introduction

Creativity can be hard to define. Despite the nebulous nature of the word, creativity has been previously defined by some researchers as “the ability to produce work that is both original (new, unusual, novel, unexpected) and valuable (useful, good, adaptive, appropriate)” [1,2]. In general, there are two main categories that comprise creative thinking: divergent thinking (the process by which new ideas are created) and convergent thinking (the process by which one evaluates the feasibility and workability of such divergent ideas) [3]. There have also been three different types of tasks, such as verbal, nonverbal, and figural, delineated to produce these divergent thinking responses.

Indeed, though it seems almost counter-intuitive to try to quantify or measure creativity, there have been many different attempts at studying and evaluating an individual’s creativity. One such method is the Torrance Test of Creative Thinking (TTCT), which was first developed In 1966 and has been used widely across the world, having been re-developed four times (1974, 1984, 1990, and 1998) and translated into 35 different languages [4]. The test has two components, verbal and figural. The figural test measures four key domains of creativity: fluidity, flexibility, originality, and elaboration. Fluidity is a measurement of the ability to produce a high number of relevant ideas as measured by the number of figures the participant is able to produce. Flexibility is a measurement of the ability to produce a wide range of variable ideas. Originality is a measurement of the number of statistically non-frequent ideas, or unique ideas. Elaboration is a measurement of the development and amount of detail that is produced around one idea [5].

The definition of dyslexia is controversial, and the origins of dyslexia are widely debated. Dyslexics have issues with spelling, word recognition, and decoding words. These deficits manifest themselves as difficulty with reading comprehension, with writing, and slower reading [6,7,8]. Despite being so common, it has been traditionally very difficult to present a clear pathologic origin for the disorder. Traditionally, dyslexia is described as a primary basic phonological processing deficit. However, more recent theories have been developed regarding difficulties with verbal short-term memory [9]. On a more global level, dyslexia is usually described as a deficit that manifests itself as a primary developmental learning disorder at the behavioral level that presents in childhood [8,10].

Outside of the behavioral model for dyslexia, others have postulated there may be a physiologic basis for dyslexia as well. For example, dyslexics have demonstrated abnormalities in visual processing, saccadic and vergence eye movements, and visual rehabilitation is widely used to improve symptoms in dyslexics [7,11,12,13,14,15]. Despite these controversies, there has never been a longitudinal or randomized control trial to further investigate these relationships between these physiologic and behavioral differences. 

There has been some data that show that dyslexics are more creative than their peers, largely in the figural categories, though it does not appear that dyslexics demonstrate higher creativity consistently in any particular domain [16,17]. For example, one study reported that dyslexics were more able to connect original thoughts to produce different types of conclusions [18]. One study reported that students with dyslexia were more creative in the originality category than their peers [19]. Our own lab has discovered that children and teenagers with dyslexia demonstrate higher creativity scores as compared to their peers, which was amplified in those who were enrolled in schools that place emphasis on visual arts [5]. Other studies have confirmed that dyslexics are more prevalent in artistic schools, perhaps demonstrating an increased aptitude for producing creative visual interpretations of the world around them [20]. It has also been shown that dyslexics tend towards careers in artistic fields, and that dyslexics have flourished in educational systems that emphasize the arts [5,20,21]. More recent studies have demonstrated that dyslexics are more creative than their peers, and that this creativity is independent of nonverbal intelligence and literary skills [22]. These findings, however, have been controversial; despite the limitations of metanalysis, some studies in dyslexics did not find any relationship between dyslexia and creativity [23,24].

Why would dyslexics be predisposed to this increased figural creativity? One answer could lie in the magnocellular theory of dyslexia, which proposes that there is a deficit in the visual magnocellular system [25]. This system is responsible for coordinating the timing of vision, particularly while reading. For example, if the eye accidentally moves the focus of vision off the fovea (a “retinal slip”), then the magnocellular system moves the eyes back to re-center the visual target on the fovea to obtain a clear visualization of the image [26]. One theory of dyslexia is that impairments in the development of the magnocellular layers of the genicular nucleus, which produce this corrective mechanism, contribute to impaired binocular fixation and poor visual localization [27]. The magnocellular theory further proposes a compensatory more powerful parvocellular visual system that might explain the higher creativity in dyslexia. Parvocells signal fine detail and color to the brain and are concentrated in the fovea [28]. Previous studies have shown that dyslexics have a higher affinity for contrast sensitivity and significantly higher blue/yellow color sensitivity, consistent with superior parocellular function [29,30,31]. As previously mentioned, it is thought that this is due to decreased magnocells with parvocellular compensation. Although this theory remains controversial, the concept of neurologic tradeoff as the basis of creativity is of interest. 

Why would an impaired visual system contribute to an increased visual creativity? Some have suggested dyslexics have an explorative bias in their cognitive search strategy that may confer an advantage [32]. Another rational hypothesis that has been proposed is related to eye movements per se. Previous studies have demonstrated that dyslexics demonstrate abnormal eye movements as compared to their peers, and that they have trouble coordinating their eyes in depth while reading and while making saccades and vergence movements to audiovisual targets [11,12,15]. We postulate that, if dyslexics’ visual and motor systems contribute to perceive the world differently (which may be associated with a difficulty reading), they may have different ways of interpreting the world, which may increase their creativity. Indeed, in a previous study conducted by the lab, dyslexics were found to have abnormal eye movements while viewing Op art pieces; it is believed that micro eye movements, particularly in depth, facilitated an illusion of movement [33]. In the same study it was shown that during these viewing periods, dyslexics perceived themselves to be more posturally destabilized than their peers, though there was no actual difference in postural parameters, suggesting dyslexics were more influenced by the perceptual illusion and had a different way of experiencing the artwork.

Given this demonstrated increased creativity in the dyslexic population, in the presence of abnormal eye movements that demonstrate dyslexics’ difficulty with keeping the eyes aligned in a sustained manner in a depth, as well as the fact that dyslexics have been shown to experience art differently, we wondered if dyslexics might interpret art with complex meanings and spaces differently. René Magritte was a Belgian surrealist artist whose work focused on the interpretation of signs, signals, representations, and meaning. His work questions the reality of objects and the physical spaces they inhabit. For example, one of his most famous paintings, entitled *La Trahison des Images*, displays a painting of a pipe with the sentence “This is not a pipe” written underneath. This particular painting forces the viewer to confront a number of questions while looking at the canvas—is it an artwork, a two-dimensional representation of a pipe, a simulacrum of a real, embodied object? Due to their specificities with eye movement instability in depth and their increased creativity scores, dyslexics’ may perceive Magritte’s artwork differently from their peers. Further, previous research has demonstrated that dyslexics’ eye movement abnormalities are particularly exacerbated while reading a nonsense text [12]. This was thought to be due to the cognitive difficulty required to decode language word-by-word. However, another hypothesis could be that the text was particularly cognitively perturbing, and that dyslexics’ eye movements are destabilized when viewing perturbing nonsense in general.

We therefore decided to evaluate dyslexics’ creativity and perception using Magritte’s complex paintings as a stimulus. We postulated our dyslexic population will demonstrate increased creativity scores as compared to their non-dyslexic peers; and that they would perceive Magritte’s pictorial ideas and spaces differently from their peers.

## 2. Materials and Methods

### 2.1. Participants

In total, 47 dyslexics (18 female, 29 male; aged 10–21; mean age 15.4) and 44 non dyslexics (22 female, 22 male; aged 8–20; mean age 14.8) participated in the study. The dyslexic adolescents attended a school specialized for dyslexic students in Paris. They were accepted to the school on the basis of their dyslexia diagnosis, though they were given their diagnosis based on extensive neuropsychologic and phonologic testing at specialized multidisciplinary centers around France. A total of 34.0% (16/47) identified their primary problem was visual/reading-based, 4.3% (2/47) was auditory, 2.1% (1/47) was writing, and 59.6 (28/47) were mixed or unknown. As is common in dyslexia, many dyslexic adolescents also reported co-morbid conditions: twelve were concurrently diagnosed with dysorthographia, dyscalcula, and/or dyspraxia. As is exceedingly common in France, 34 participants had been to orthoptic rehabilitation or had seen an orthoptist. All participants had no neurologic or psychiatric abnormalities; non-dyslexics had no deficiencies in reading, writing, vision, or visual impairment. The investigation adhered to the principles of the Declaration of Helsinki and was approved by our Institutional Human Experimentation Committee (CPP CNRS 18 011). Written, informed consent was obtained from the adolescents and/or their parents after they were given an explanation about the experimental procedure. The tests were conducted by two research assistants, who were trained together using the same material and conducted the experiment together for each measurement.

### 2.2. Eye Movement Recording Device

Eye movements were recorded binocularly with a head-mounted video-oculography device, Pupil Core, with recording at 200 Hz in binocular vision with an accuracy of 0.60° and precision of 0.02° (Pupil Labs, Berlin, Germany).

### 2.3. Calibration of the Pupil Labs Device

The standard Pupil Labs calibration (Pupil Capture) was applied using a target that was presented at a viewing distance of 1m. The subject fixated on the center of the target and moved their head rightward, downward, leftward, and upward at their own pace. They then repeated the sequence.

### 2.4. Creativity Assessment

**Figure 1:**
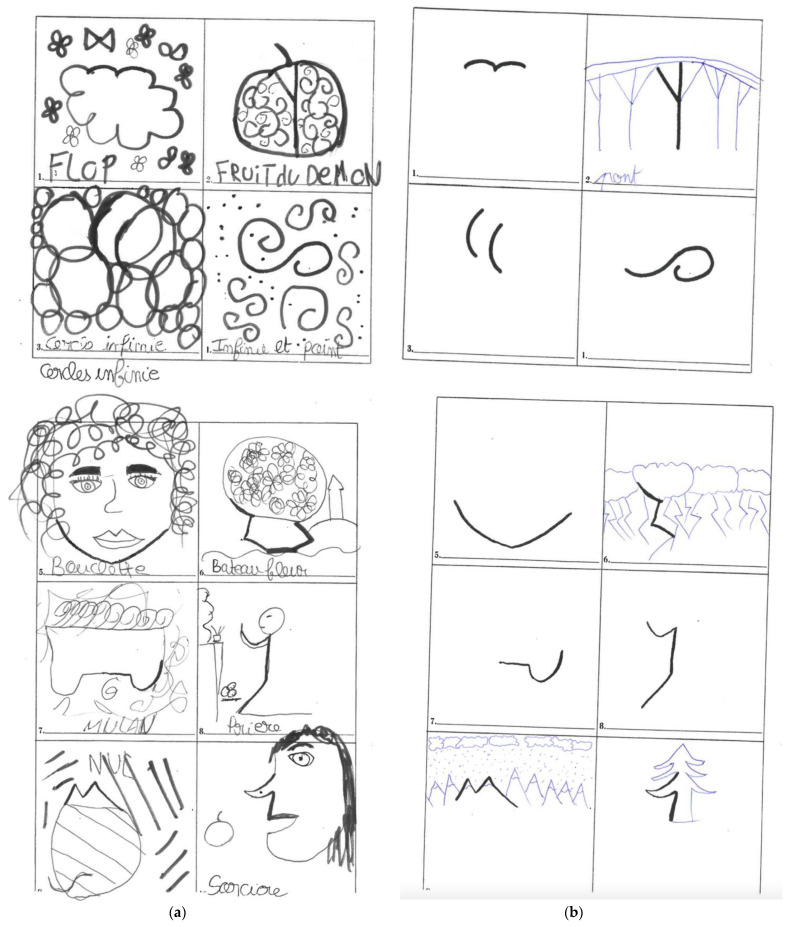
(**a**) Example of a dyslexic response with high fluidity (score of 10) and high flexibility (score of 10) to Part 2 of the figural portion of the Torrance Test for Creative Thinking. (**b**) Example of a non-dyslexic response with low fluidity (score of 3) and low flexibility (score of 3) to Part 2 of the figural portion of the Torrance Test for Creative Thinking.

The Torrance Test of Creative Thinking (TTCT)-Figural form is an age-normed test (up to 18 years old) [4]. It has been widely used across different countries for over 50 years and translated into multiple different languages, including French. The TTCT also has been previously used in medical research; for example, Richard Levy used the TTCT to examine creativity in patients with frontotemporal dementia [34]. The TTCT also confers a particular advantage in assessing the dyslexic population given their difficulty with textual deconstruction in that it offers a figural component to assessing creativity. Other methods of assessing creativity, such as those that examine divergent and convergent thinking, were considered, but ultimately the TTCT was chosen because we believe it offers a nuanced examination of creative parameters through a figural challenge. It is a thirty-minute test consisting of three parts of ten minutes each. Each component asks the participant to produce an unusual drawing from standardized shapes that were the same for each participant (see Figure 1a,b). The TTCT-Figural form is scored to assess four different components of creativity: originality (how uncommon each drawing is); fluency (the number of relevant drawings produced); elaboration (how enriched the drawings are); and flexibility (the number of different ideas created by the drawings). Each 10 min component was scored, which was then converted into a standard score with a standardized TTCT chart. Each participant was administered the TTCT-Figural form in a quiet room. Each participant was instructed according to the TTCT protocol exactly as it is written. The test was conducted with paper and pencil. The results were scored by the investigators who were trained in the analysis of the Torrance test. The investigators were blinded to their classification as being dyslexic or non-dyslexic.

### 2.5. Procedure

The same instructions were given for each participant. Participants were asked to stand in front of a laptop that was positioned so that the center would be 40 cm away from their eyes at eye-level. The image was positioned to be in the middle of their vision with the center of the screen at eye level. Each participant was instructed to keep their head still and to not move their body during testing. They were then asked to fixate on a target at the bottom right corner of the computer screen prior to being shown each painting. Each participant was then invited to explore the image as they wished for thirty seconds when the image appeared. They were then shown each image on a black background sequentially for 30 s. In between each painting they were given 30 s to rest prior to the next viewing session, during which they looked at a black screen. They viewed three paintings in a row by René Magritte: La trahison des images (1929); La condition humaine (1933); and L’art de la conversation (1950). Each participant viewed the paintings in this order without variation.

### 2.6. Paintings

The three paintings presented to dyslexics were strategically chosen. In a previous study conducted by the lab, it was found that dyslexics had more difficulty with binocular coordination while reading a more complex, nonsense text than when they were reading an easier text that carried a narrative. Indeed, another study has demonstrated that predictive power of determining dyslexia was stronger while studying more difficult texts [35]. We therefore concluded that dyslexics’ oculomotor profiles were destabilized while reading more complex nonsense texts. We therefore wanted to demonstrate nonsense visually to the dyslexic children in lieu of a textual nonsense. We chose three complex paintings by Magritte with layers of meaning that is contradictory, impossible, and that are difficult to reconcile. Magritte, a surrealist artist, embodies the essence of surrealism, painting the absurd and the incongruent. We hypothesize that these complicated paintings will act similarly to nonsense text and provoke unstable eye movements in the dyslexic population.

**Figure 2:**
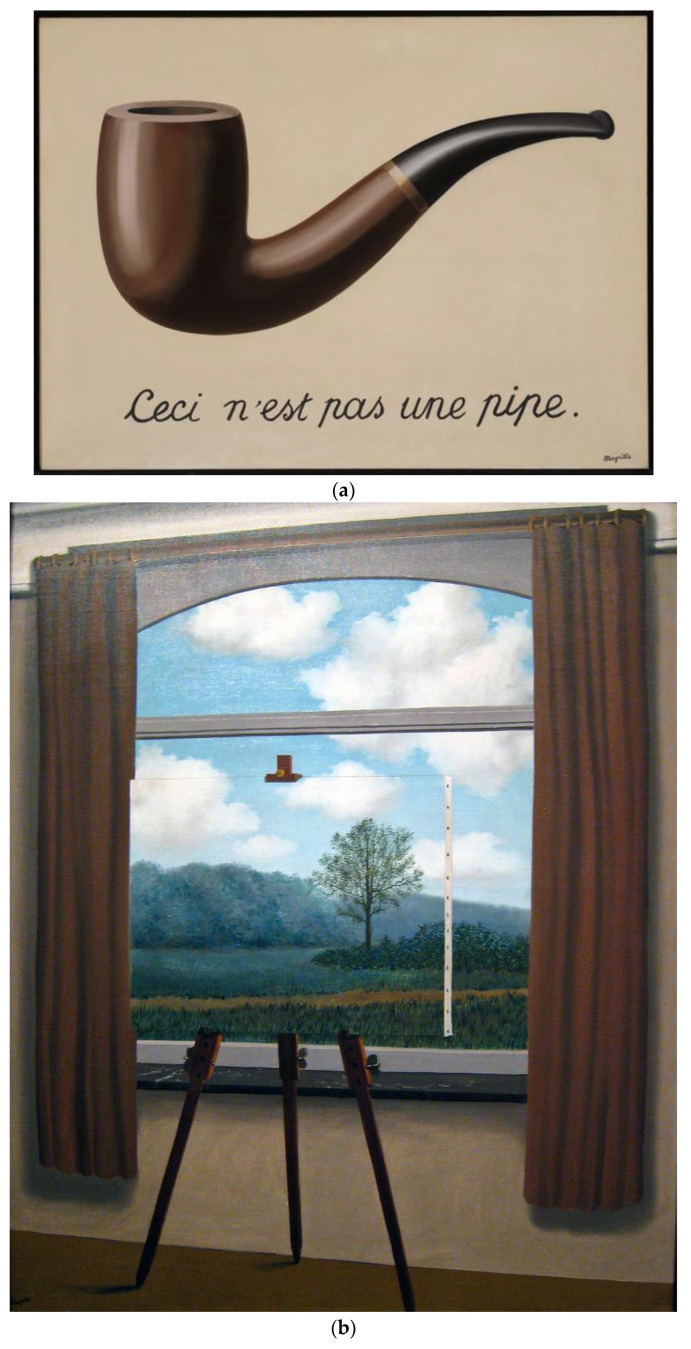

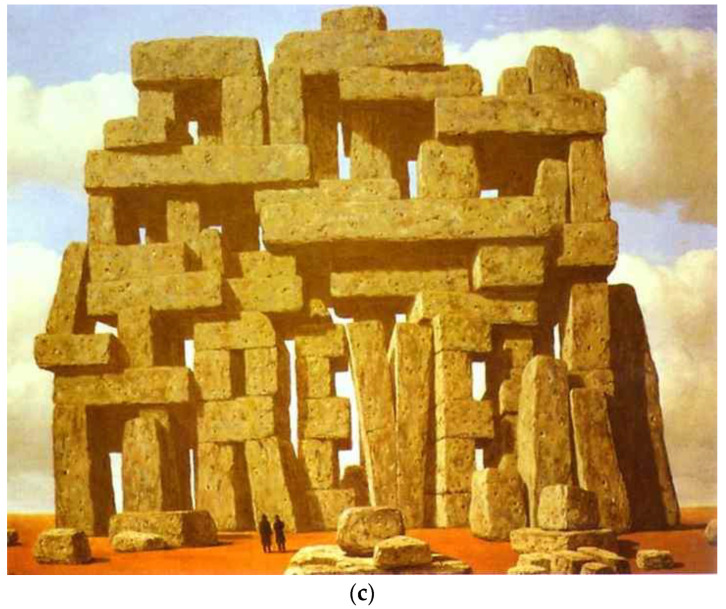
(**a**) Painting 1: René Magritte, *La Trahison des Images*, 1929. (**b**) Painting 2: René Magritte, *La Con-dition Humaine*, 1933. (**c**) Painting 3: René Magritte, *L’Art de la Conversation*, 1950.

The first painting, *La Trahison des Images*, 1929, was chosen because, at first glance, it appears to demonstrate a direct contradiction between image and message (Figure 2a). While the painting depicts a pipe, it also depicts a sentence underneath the pipe that reads “Ceci n’est pas une pipe [This is not a pipe]”. The adolescents would therefore have to process the apparent contradiction between image and text and interpret the layers of meaning behind the painting. 

The second painting, entitled *La Condition Humain*, 1933, depicts an easel that displays a painting that directly matches the backyard background it is placed in front of (Figure 2b). It is difficult at first to interpret the painting as being separate from the room in which the easel is placed from the backyard the room looks out onto. The challenge for adolescents in viewing this painting was to interpret the easel as holding a painting, which is a two-dimensional surface separate from the three-dimensional space that the two-dimensional painting is depicting. 

Finally, the third painting, entitled *L’Art de la Conversation*, 1950, depicts two small figures standing in front of a rock formation (Figure 2c). The rocks form the word *REVE* (dream). Given their difficulty with reading, we were curious to see if dyslexic adolescents would have difficulty picking out the letters in the two-dimensional complicated space.

### 2.7. Subjective Evaluation of the Paintings

After viewing the paintings, each participant completed a questionnaire surveying their subjective responses to each of the paintings. While they were filling out the survey, they could review each painting on the laptop. They were asked how they would rate each painting on a scale from 1 to 10 (1 = you do not like it at all; 10 = you love it) and rate how bizarre they found each painting (1 = not at all; 10 = very bizarre). 

### 2.8. Data Analysis

AIDEAL (PCT/EP2021/062224 7 May 2021), a software developed at ORASIS-EAR, was used to analyze data recorded with the Pupil Labs eye tracker. To analyze the eye movements, AIDEAL calculated the conjugate signal, i.e., the L+R eye position/2. The saccade was defined as the time points where the peak velocity went above or below 10% of the peak velocity; this corresponded to values above or below 40°/s. AIDEAL defined the average velocity as the ratio of the total amplitude in degrees/time in seconds. The disconjugacy during saccades, or binocular coordination, was measured as the difference in amplitude between the left and right eye signal. The difference in drift amplitude in the first 80 or 160 ms of fixation was calculated as the disconjugate drift. Eye movements with blinks or artifacts (defined as values beyond physiologic parameters; e.g., short fixation durations of <100 ms, small saccade amplitudes of <0.5 degrees, and long fixations (>700 ms) were automatically discarded by AIDEAL.

### 2.9. Statistical Analysis

The Shapiro–Wilk test was performed for each comparison and none of the data were found to be normally distributed. As such, we performed the non-parametric Mann–Whitney U test for means comparison for creativity scores between the dyslexic and non-dyslexic populations.

In a second analysis, given that the data were not normally distributed, we performed the non-parametric Mann–Whitney U test to compare dyslexic and non-dyslexic eye movements as they looked at each painting. In terms of subjective ratings, again, as the data were not normally distributed, we performed the non-parametric Mann–Whitney U test for comparing subjective ratings of appreciation and bizarreness between the dyslexic and non-dyslexic population as they looked at each painting. 

In a third analysis, we calculated the Spearman’s rho correlation between the subjective responses while viewing each painting and the creativity scores in the dyslexic population. 

For all analyses, the statistical significance was set at *p* ≤ 0.05. We did not attempt to correct for multiple comparisons. All analyses were performed using SPSS version 25 (IBM Corp. Released 2017. IBM SPSS Statistics for Windows, Version 25.0. Armonk, NY, USA: IBM Corp.).

## 3. Results

### 3.1. Creativity Scores

Dyslexics were found to have higher creativity scores in the fluidity (9.68 vs. 8.75; *p* = 0.013) and flexibility (8.26 vs. 7.30, *p* = 0.031) categories. They were not found to be statistically more creative in the other domains as compared to their non-dyslexic peers (see Table 1).

### 3.2. Eye Movement Differences between Groups by Painting

For all three paintings, dyslexics demonstrated a higher duration of saccades, a faster peak velocity, and a slower average velocity in saccades made to the left and right while viewing (see Table 1, Table 2 and Table 3). This oculomotor profile is similar to that found in dyslexics while reading and while making saccades to audiovisual targets, as well as while viewing Op art artworks [11,12]. They also demonstrated a higher disconjugacy during saccades to the right and left while viewing all paintings. Finally, dyslexics demonstrated a lower amplitude during right and left saccades while viewing all paintings, meaning they explore the painting with smaller saccades.

While viewing Painting 3, dyslexics demonstrated a longer fixation disconjugacy after the saccade as compared to non-dyslexics (0.88 +/− 0.46 vs. 0.69 +/− 0.33; *p* = 0.033).

**Figure 3:**
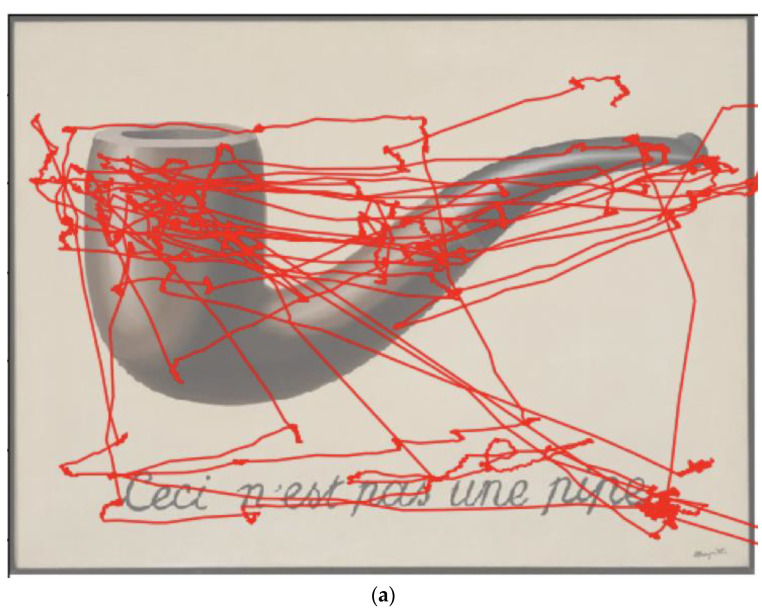

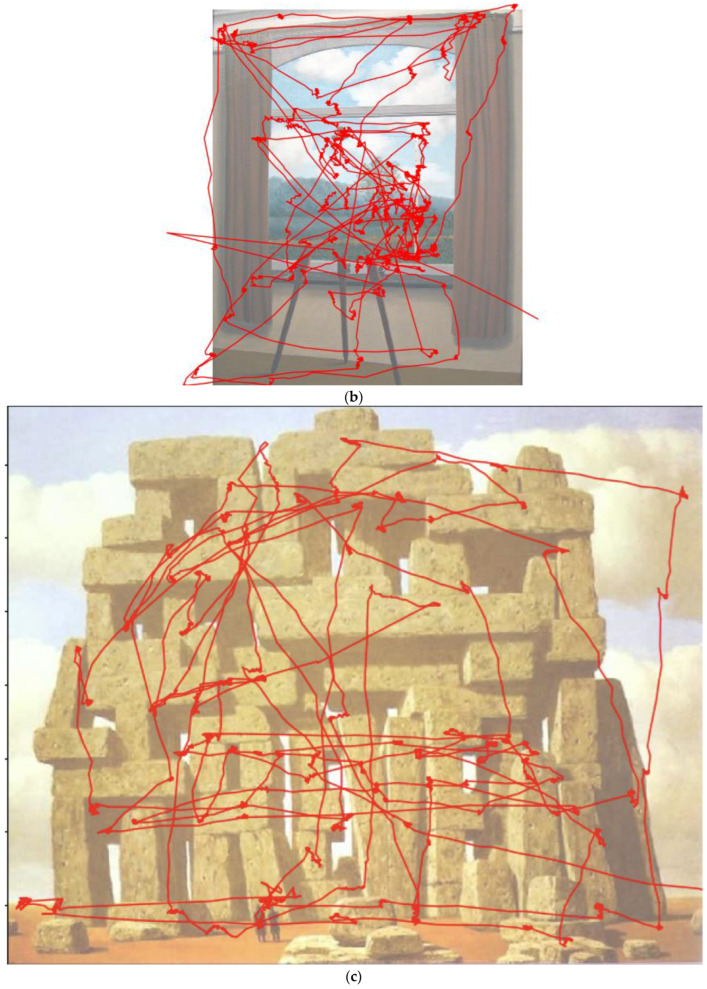
(**a**) Example of one dyslexic participant’s eye tracing over the 30 s viewing period. (**b**) Example of one dyslexic participant’s eye tracing over the 30 s viewing period. (**c**) Example of one dyslexic participant’s eye tracing over the 30 s viewing period.

Please see Figure 3a–c for an example of an individual dyslexic’s eye movements during the thirty-second trial overlayed over each painting.

### 3.3. Subjective Reports of Appreciation and the Perception of the Bizarre

**Figure 4:**
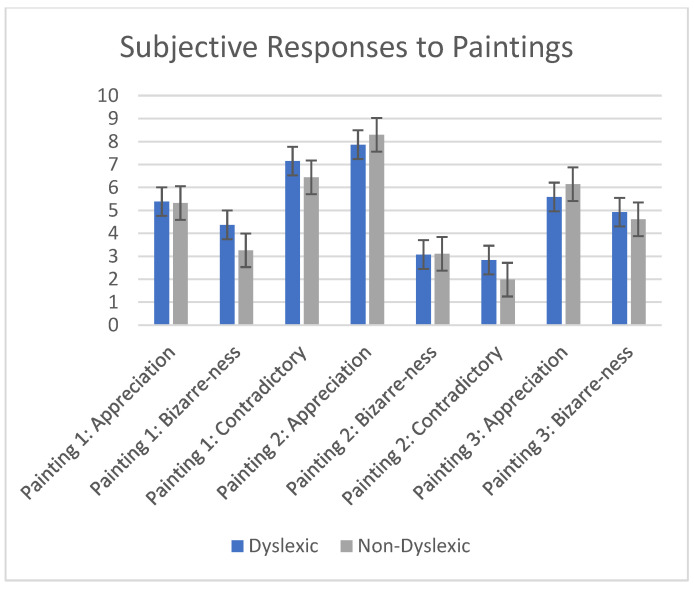
Subjective reports of appreciation, perception of the bizarre, and contradiction in each population.

There were no significant differences in subjective ratings of appreciation, bizarreness, or how contradictory the paintings were between the two populations (See Figure 4). 

**Figure 5:**
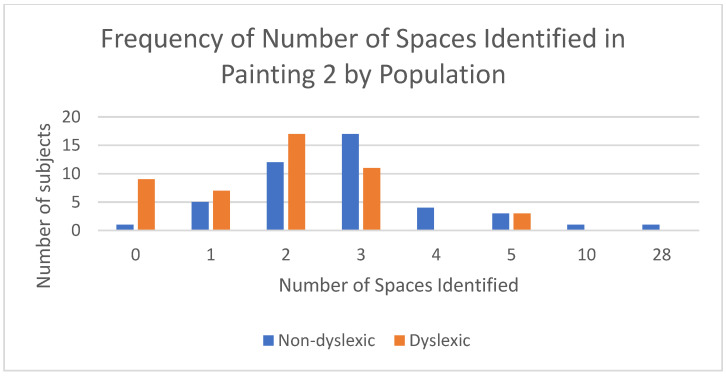
Number of spaces perceived in Painting #2 by population.

While viewing Painting 2, participants were asked how many spaces they perceived to be represented in the painting. Non-dyslexics reported perceiving significantly more spaces than dyslexics (1.96 +/− 1.33 vs. 3.44 +/− 4.17; *p* = 0.003; Figure 5). From their comments, dyslexics seemed to identify two spaces in the painting: either the inside and outside or the outside and the painting itself. Non-dyslexics seemed to identify three spaces more frequently: inside, outside, and the painting itself. 

**Figure 6:**
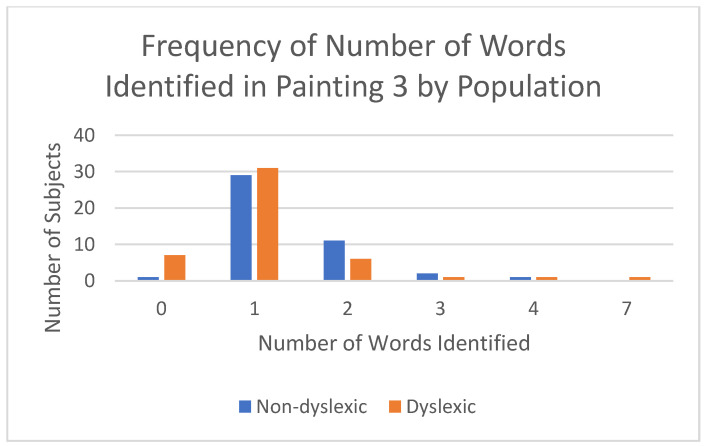
Number of words perceived in Painting #3 by population.

While viewing Painting 3, participants were asked how many words they recognized in the painting. Non-dyslexics recognized significantly more words as compared to dyslexic participants (1.23 +/− 1.13 vs. 1.38 +/− 0.73; *p* = 0.043; Figure 6). Dyslexics more frequently identified one word “Reve”, but sometimes identified an extra letter or word. Non-dyslexics more frequently identified extra words or letters in addition to identifying the correct word “Reve”. 

### 3.4. Correlation between Appreciation and the Perception of the Bizarre

For both populations, the more the adolescents found the paintings to be contradictory, the less they appreciated the artwork (dyslexic: correlation co-efficient −0.247, *p* < 0.017; non-dyslexic: correlation co-efficient −0.410; *p* < 0.001). 

## 4. Discussion

In terms of creativity, in our population, dyslexics were found to be more creative than non-dyslexics in terms of fluidity and flexibility. Fluidity is the measurement of the number of relevant ideas an adolescent produced in response to a single prompt. Flexibility is the ability to produce a wide range of ideas to a particular stimulus. Our study confirms that dyslexics exhibit higher creativity scores in terms of flexibility and fluidity [5]. Unlike previous studies, the current study examined adolescents instead of children. Perhaps, as dyslexics learn to operate in the regimented world of the educational system, they struggle and adapt to see things differently from their peers by becoming more flexible and producing a greater number of ideas to overcome an issue. Creativity may also depend on the population of the particular group of adolescents who participated. The dyslexic adolescents all attended a school that specialized in educating dyslexic children. The non-dyslexic population was recruited from private schools that had also granted their students access to many diverse resources. Therefore, one could say they each had privileged educations. In any case, it is useful to know that dyslexic adolescents demonstrate higher creativity scores specifically in flexibility and fluidity, though a higher creativity may also be influenced by educational background as previously reported by Kapoula et al. [5].

From an eye movement perspective, once again, dyslexics demonstrated an abnormal pattern of eye movement as they viewed all three paintings as compared to their non-dyslexic peers. This uncoordinated eye movement pattern, represented by a higher saccade disconjugacy, a higher duration, and a slower average velocity despite a faster peak velocity, was similar to those they produced while looking at Op art, while viewing audiovisual targets, and while reading. For further discussion of the physiologic basis for potential differences in binocular coordination, please see Ward and Kapoula 2020, 2021, and 2022 [11,12,36]. These eye movements appear to be the same no matter what the task is. Even in these less regimented, playful conditions such as regarding paintings, there are eye movement differences that persist despite the task, which could represent a physiologic oculomotor signature of dyslexia. This further consolidates evidence for fundamental differences in eye movements between dyslexics and non-dyslexics. It should be noted, however, that the paintings were chosen as specific stimuli, because they challenge perception in different ways. Similar to reading nonsense text, viewing these paintings are cognitively challenging, with an additional layer of perceptual challenge. These paintings present conditions that dyslexics are sensitive to, and their eye movements reveal once again their oculomotor instability.

Despite their differences in creativity and in eye movement profile, dyslexics and non-dyslexics did not subjectively perceive the paintings differently in terms of appreciation, how bizarre, and how contradictory the pieces were. Interestingly, however, there were some significant differences in the perception of space and words. While viewing Painting 2, dyslexics reported perceiving significantly fewer spaces in the painting as compared to their peers.

As discussed, it has been previously shown that dyslexics may have difficulty with depth perception, both while viewing targets in space, while reading, and while viewing two-dimensional representations of the illusion of depth in Op art paintings. Because of their poor binocular coordination (i.e., disconjugacy) and eye drift during fixation, it follows that dyslexics’ visual world could appear more blurry and unstable. This difficulty with depth perception is again represented while viewing this trompe l’oeil image in which multiple spaces are depicted in depth. We postulate that dyslexics’ difficulty to interpret this two-dimensional representation of a complex three-dimensional space may again be related to their difficulty coordinating their eyes in depth. This representation is slightly different from previous descriptions of dyslexics’ interaction with space in that it requires the adolescent to recognize layers of conceptual space. Whereas our previous research has focused on examining eye movements while dyslexics view objects in true three-dimensional space or during the illusion of three-dimensional space, Magritte’s paintings force the viewer to confront a conceptual space represented by a painting in a painting that is difficult to discern from the background of the painting. Conceptual space perception is different from the illusion of depth that is triggered by Op art, which targets lower visual and eye movement processing, in that it is perhaps a more complex, subtle test. It is interesting that this study demonstrates that dyslexic’s abnormal oculomotor profile persists despite this difference in depth perception, suggesting a possible a physiologic oculomotor signature of dyslexia for all the stimuli selected.

An alternative explanation of this result may be that the dyslexics reported the reality of the two major spaces: inside vs. outside. Indeed, they did answer more frequently that they perceived two spaces: inside and outside more frequently than non-dyslexics, who more frequently reported perceiving three spaces: inside, outside, and the space of the painting. It is interesting that dyslexics perceived depicted reality and did not consider the third space of the painting as frequently as non-dyslexics. Perhaps dyslexics are more pragmatic, while non-dyslexics are more open to consider surreal, illusory spaces. As not every subject freely elaborated on which spaces they perceived, further questioning and more focused spatial analysis of the eye scan paths could provide more objective information regarding their areas of interest. 

Dyslexics also reported that they recognized significantly fewer words while viewing Painting 3. Painting 3 has one word represented, “REVE”, which means “dream” in French. Despite there only being one word, non-dyslexic adolescents reported recognizing more than one word more frequently than dyslexics; and dyslexics reported not recognizing any words more frequently than non-dyslexics. What is curious, however, is that non-dyslexic students tended to find more words than were actually represented in the painting, demonstrating an increased propensity to find different words that are not actually represented. Though they came up with more words, non-dyslexics were not actually found to be more creative than dyslexics. The explanation for this may lie in the type of creativity testing the subjects undertook: all subjects participated in the figural form of the TTCT, which tests for visual creativity. We did not measure creativity with the verbal form of the TTCT. Future studies should test verbal creativity between dyslexic and non-dyslexic populations to see if dyslexics if there is any relationship between verbal creativity and reading difficulty.

Another alternative explanation is that dyslexics’ difficulties with visual coordination impact their ability to confabulate. As the current and previous studies have shown, dyslexics’ eye movements are physiologically perturbed by any visual challenge. Perhaps the challenge of uncovering pictorial illusions bears too high of a cognitive load to look for further hidden words or spaces in the dyslexic population, while non-dyslexics are more free to confabulate.

These differences in space and word perception are puzzling. Given that dyslexics were shown to be more creative on psychometric testing, one would expect dyslexics to note more spaces and more words in the pictures. It may be that other aspects of the dyslexic adolescent (difficulty with word and depth perception in particular) limit the perception of additional words and spaces in depth. On the other hand, one could consider that dyslexics may be more in tune with the artist’s represented trompe l’oeil image and are less likely to confabulate additional spaces or words beyond what the artist represented. 

For both populations, the more adolescents found the paintings to be contradictory, the less they liked the artwork, perhaps indicating that visual conflict is less aesthetically pleasing. However, both populations appeared to like Paintings 2 and 3 more when they uncovered a hidden space or the hidden word; as all adolescents tended to like the paintings more when they appreciated more spaces or words, indicating some kind of pleasure to solve a visual puzzle or uncover a hidden meaning. 

There are limitations to our study. First, we were unable to further delineate specific eye movements while looking at specific portions of each painting. We instead averaged each eye movement parameter over the thirty-second viewing period. We therefore are not able to determine any differences in eye movement by group that could be associated with areas of high visual interest or conflict in each piece. For these more figural paintings that facilitate more perceptual and conceptual challenges, more specific modulation of eye movement parameters would be helpful, in order to target specific changes in movement or to highlight visual interest regarding where and how they view different parts of the painting. Future studies and new paradigms analyzing eye movements simultaneously over time and space, with recognition of time spent decoding wording would be of interest. Similarly, goal-directed studies, i.e., asking an adolescent to identify and point with their eyes at the most ambiguous part of the painting, would also be of interest. Future studies should aim to analyze eye movements in particular key high-interest or high-conflict areas of the painting, which may uncover a more subtle analysis of the way subjects view these complex pieces; additional studies are currently in progress. 

Finally, it is important to consider the populations studied in this experiment. We did not compare IQ or emotional intelligence between the two subject groups, which may have contributed to differences in how each group interacted with the artwork. The dyslexic population was recruited from a school for dyslexic children in Paris, while the control population was recruited from a private school in Paris. Both groups of adolescents came from highly advantaged backgrounds, in both schooling and family support. This may have influenced the way they interacted with the artwork and their subjective responses to the works. Indeed, research from our own lab has demonstrated the effect of education on creativity [5]. In this case, however, it is important to note that both groups were recruited from advantaged, though different, schooling and backgrounds. Therefore, we believe these populations are worth comparison. 

## 5. Conclusions

For the first time to our knowledge, dyslexic adolescents (mean age 15.4 years old) were demonstrated to be more creative in terms of flexibility and fluidity than their non-dyslexic peers. Both populations came from privileged social backgrounds and attended schools with strong educational backgrounds and objectives. This represents a difference from previous studies, which have found dyslexic children to be more creative in all domains. Dyslexics also perceived the paintings differently from non-dyslexics. Indeed, we found that dyslexics perceived fewer conceptual spaces and fewer words than their non-dyslexic peers, which was puzzling. We postulate this could be either related to their uncoordinated eye movement abnormalities or to a perception of the painter’s specific conveyed tromp d’oeil or illusions without confabulation.

Dyslexics also demonstrated the same abnormal oculomotor profile, in that they demonstrated difficulty with binocular coordination and abnormalities in their velocity profile, which was sustained throughout all viewing conditions. Perhaps artistic stimuli such as Magritte’s paintings represent a similar challenge to reading a non-sense text, which exacerbates dyslexics’ eye movement abnormalities. This susceptibility could be a biomarker of their sensitivity to nonsense, to contradiction, and to reality versus illusion.

These findings are consistent with multiple different theories on the origins of dyslexia, which point to a more nuanced definition of dyslexia than is previously discussed in the literature. There are different functional aspects in the way that dyslexics move their eyes, react to nonsense, and in the way they perceive. Beyond considering them as a deficient population defined by either their difficulty reading, their poor eye movements, or their trouble learning, we should more holistically revisit what it means to be dyslexic and reconsider their differences as potentially beneficial.

Our analysis is focused on physiologic parameters of eye movements rather than on localization. A companion paper (El Hmimdi et al. under press) indicates the predictive power of such analysis in discriminating between dyslexics and non-dyslexics that also reflects differences in their creativity. Finally, future studies should focus on new ways and paradigms to evaluate and appreciate the potential physiologic connection between dyslexia and creativity outside of conventional testing such as the TTCT. Perhaps, beyond psychometric evaluation of creativity, the eye movement specificities in interplay with such challenging artwork in connection with verbal reports to questionnaire is another sensitive way to assess aesthetic sensitivity and creative expression. 

## 6. Patents

Zoi Kapoula has applied for patents for the technology used to conduct this experiment: AIDEAL: PCT/EP2021/062224 7 May 2021, patent application pending EP22305903.1.

## Figures and Tables

**Table 1:** Eye movements while viewing Painting 1.

	Dyslexic			Non-Dyslexic			*p*-Value
	Median	SD	N	Median	SD	N	
**Left Amplitude (deg)**	**3.56**	**0.99**	**2828**	**4.22**	**0.74**	**2918**	**<0.001**
**Right Amplitude (deg)**	**3.45**	**0.94**	**3116**	**4.04**	**0.74**	**3223**	**0.004**
**Left Duration (ms)**	**84.23**	**45.39**	**3526**	**68.00**	**38.50**	**3633**	**0.004**
**Right Duration (ms)**	**86.67**	**62.56**	**3711**	**61.10**	**32.06**	**3832**	**<0.001**
**Left Peak Velocity (deg/s)**	**188.86**	**74.96**	**3507**	**146.41**	**59.51**	**3613**	**<0.001**
**Right Peak Velocity (deg/s)**	**173.92**	**77.24**	**3717**	**121.72**	**48.92**	**3835**	**<0.001**
**Left Average Velocity (deg/s)**	**87.10**	**36.93**	**3554**	**124.39**	**35.01**	**3660**	**<0.001**
**Right Average Velocity (deg/s)**	**81.87**	**31.66**	**3725**	**121.00**	**32.64**	**3846**	**0.003**
Left Fixation Disconjugacy 80 ms after saccade (deg)	0.62	0.24	3524	0.64	0.22	3630	0.93
Right Fixation Disconjugacy 80 ms after saccade (deg)	0.58	0.21	3714	0.55	0.18	3832	0.37
Left Fixation Disconjugacy 160 ms after saccade (deg)	0.95	0.41	3537	0.91	0.43	3641	0.50
Right Fixation Disconjugacy 160 ms after saccade (deg)	0.87	0.80	3702	0.81	0.84	3821	0.39
**Left Disconjugacy During Saccade (deg)**	**2.76**	**3.20**	**3500**	**1.61**	**0.84**	**3605**	**0.037**
**Right Disconjugacy** **During Saccade (deg)**	**2.61**	**3.43**	**3707**	**1.26**	**0.71**	**3828**	**0.002**
Left Fixation Duration (ms)	288.17	75.54	2223	315.51	92.60	2291	0.20
Right Fixation duration (ms)	286.45	72.00	2415	307.69	79.29	2495	0.19

**Table 2:** Eye movements while viewing Painting 2.

	Dyslexic			Non-Dyslexic			*p*-Value
	Median	SD	N	Median	SD	N	
**Left Amplitude (deg)**	**2.84**	**0.76**	**2127**	**3.33**	**0.90**	**2195**	**0.010**
**Right Amplitude (deg)**	**2.83**	**0.75**	**2063**	**3.25**	**0.90**	**2134**	**0.03**
**Left Duration (ms)**	**101.23**	**67.97**	**2473**	**69.83**	**45.73**	**2542**	**0.001**
**Right Duration (ms)**	**98.70**	**98.83**	**2386**	**67.93**	**38.26**	**2465**	**0.006**
**Left Peak Velocity (deg/s)**	**172.90**	**108.43**	**2464**	**119.17**	**56.25**	**2536**	**<0.001**
**Right Peak Velocity (deg/s)**	**165.01**	**77.30**	**2383**	**124.71**	**82.54**	**2462**	**<0.001**
**Left Average Velocity (deg/s)**	**64.93**	**33.41**	**2452**	**93.85**	**34.93**	**2524**	**<0.001**
**Right Average Velocity (deg/s)**	**67.97**	**27.76**	**2399**	**94.32**	**32.13**	**2478**	**<0.001**
Left Fixation Disconjugacy 80 ms after saccade (deg)	0.65	0.40	2464	0.55	0.20	2536	0.22
Right Fixation Disconjugacy 80 ms after saccade (deg)	0.64	0.47	2383	0.57	0.27	2460	0.39
Left Fixation Disconjugacy 160 ms after saccade (deg)	0.97	0.70	2461	0.75	0.29	2533	0.15
Right Fixation Disconjugacy 160 ms after saccade (deg)	1.03	0.93	2394	0.79	0.35	2472	0.11
**Left Disconjugacy During Saccade (deg)**	**2.66**	**2.79**	**2458**	**1.31**	**0.72**	**2528**	**0.001**
**Right Disconjugacy** **During Saccade (deg)**	**2.44**	**2.40**	**2383**	**1.41**	**1.03**	**2462**	**0.003**
Left Fixation Duration (ms)	320.76	89.34	1275	289.87	90.04	1309	0.11
Right Fixation duration (ms)	332.38	70.14	1236	308.22	114.63	1285	0.41

**Table 3:** Eye movements while viewing Painting 3.

	Dyslexic			Non-Dyslexic			*p*-Value
	Median	SD	N	Median	SD	N	
**Left Amplitude (deg)**	**3.22**	**0.78**	**2753**	**3.78**	**0.74**	**2827**	**<0.001**
**Right Amplitude (deg)**	**3.10**	**0.77**	**2884**	**3.78**	**0.70**	**2978**	**<0.001**
**Left Duration (ms)**	**85.27**	**61.07**	**3161**	**57.42**	**29.36**	**3246**	**<0.001**
**Right Duration (ms)**	**75.98**	**33.07**	**3196**	**56.03**	**31.07**	**3300**	**<0.001**
**Left Peak Velocity (deg/s)**	**159.41**	**75.45**	**3146**	**114.94**	**49.11**	**3234**	**<0.001**
**Right Peak Velocity (deg/s)**	**147.49**	**72.37**	**3200**	**107.43**	**50.60**	**3303**	**<0.001**
**Left Average Velocity (deg/s)**	**77.52**	**28.21**	**3177**	**107.24**	**29.89**	**3264**	**<0.001**
**Right Average Velocity (deg/s)**	**75.58**	**27.16**	**3216**	**103.35**	**28.99**	**3319**	**<0.001**
Left Fixation Disconjugacy 80 ms after saccade (deg)	0.62	0.31	3189	0.55	0.18	3276	0.65
Right Fixation Disconjugacy 80 ms after saccade (deg)	0.54	0.20	3229	0.50	0.23	3332	0.20
Left Fixation Disconjugacy 160 ms after saccade (deg)	0.93	0.55	3185	0.77	0.29	3272	0.34
**Right Fixation Disconjugacy 160 ms after saccade (deg)**	**0.88**	**0.46**	**3233**	**0.69**	**0.33**	**3338**	**0.033**
**Left Disconjugacy During Saccade (deg)**	**2.51**	**2.93**	**3167**	**1.32**	**0.83**	**3252**	**0.005**
**Right Disconjugacy** **During Saccade (deg)**	**2.33**	**2.74**	**3212**	**1.20**	**0.74**	**3315**	**0.012**
Left Fixation Duration (ms)	317.29	72.50	1885	337.39	77.11	1932	0.187
Right Fixation duration (ms)	336.97	61.90	1989	345.70	65.93	2048	0.634

## Data Availability

The datasets generated during and/or analyzed during the current study are available from the corresponding author on reasonable request.
